# Distilling the curriculum: An analysis of alcohol industry-funded school-based youth education programmes

**DOI:** 10.1371/journal.pone.0259560

**Published:** 2022-01-12

**Authors:** May C. I. van Schalkwyk, Mark Petticrew, Nason Maani, Ben Hawkins, Chris Bonell, Srinivasa Vittal Katikireddi, Cécile Knai

**Affiliations:** 1 Faculty of Public Health and Policy, London School of Hygiene & Tropical Medicine, London, United Kingdom; 2 SPECTRUM Consortium (Shaping Public Health Policies to Reduce Inequalities and Harm), London, United Kingdom; 3 MRC/CSO Social and Public Health Sciences Unit, University of Glasgow, Glasgow, United Kingdom; George Institute for Global Health, INDIA

## Abstract

**Background and aim:**

For decades, corporations such as the tobacco and fossil fuel industries have used youth education programmes and schools to disseminate discourses, ideas and values favourable to their positions, and to pre-empt regulation that threatens profits. However, there is no systematic research into alcohol industry-funded youth education programmes. This article serves to address this important gap in the literature.

**Methods:**

Using a discourse theoretical approach informed by poststructural discourse theory and critical discourse analysis, we analysed teaching materials from three school-based youth education initiatives which focus on alcohol consumption and health harms: Drinkaware for Education, The Smashed Project (funded by Diageo), and Talk About Alcohol (Alcohol Education Trust). These materials, some of which are disseminated internationally, are provided to schools through intermediary bodies in receipt of alcohol industry funding.

**Findings:**

The analysis found that these materials drew from and presented discourses of personal responsibility, moderate alcohol consumption, and involved a narrowing of the problem definition and causes. The locus of the problem is located by the discourses within individuals including youth, with causes of youth alcohol consumption repeatedly presented as peer pressure and ‘poor choices’, with little or no mention of alcohol industry marketing or other practices. All programmes promoted familiarisation and normalisation of alcohol as a ‘normal’ adult consumer product which children must learn about and master how to use responsibly when older. The discourses constructed in these materials closely align with those of other alcohol industry corporate social responsibility discourses which employ selective presentation of harms, including misinformation about cancer, and ambiguous terms such as “responsible drinking”. Furthermore, the role of alcohol price, availability and access, and the impacts of alcohol and the industry on inequities were not articulated within the discourses. The research was limited to an analysis of teaching materials and further research is needed to explore their impact on youth, teachers and wider discourses and social norms.

**Conclusion:**

Alcohol industry-sponsored youth education programmes serve industry interests and promote moderate consumption while purportedly educating children about harms and influences of alcohol use. There are considerable conflicts of interest in the delivery of alcohol education programmes funded by the alcohol industry and intermediary bodies in receipt of such funding. Alcohol education materials should be developed independent from industry, including funding, and should empower children and young people to understand and think critically about alcohol, including harms and drivers of consumption, and effective interventions needed to protect them and others from alcohol-related harms. Independent organisations can use this analysis to critique their materials to strengthen alignment with meeting student and public health interests. The ongoing exposure of children and young people to such conflicted and misleading materials needs urgent attention from policymakers, practitioners, teachers and parents, and resources dependent on industry support should cease being used in schools.

## Introduction

The alcohol industry, like other industries that produce harmful products, employs a range of marketing and corporate political strategies to advance their interests [1–3], including accessing and influencing youth and schools. Among other practices, corporate entities develop, sponsor, and promote youth prevention and education programmes which they claim are intended to prevent and teach about public health issues such as under-age smoking and alcohol use, and childhood obesity [4–6]. Such activities allow corporations to frame themselves as responsible corporate citizens who are “part of the solution” not the problem [7, 8]. The development and delivery of such programmes facilitates the establishment of partnerships with reputable third parties and gaining access to policy-makers, children, teachers and families [5–7, 9–23]. This is favourable to industry interests as it can serve to build brand awareness and perceptions of positive corporate citizenship deflecting away from negative corporate practices, as well as aiding in maintaining, normalising, and disseminating corporate-friendly ideas, values, and discourses to children, youth, teachers and parents. As Molnar explains:

Corporately sponsored educational materials give students corporately sanctioned points of view on a wide range of topics and serve as opportunities to make ‘brand impressions’ with the school as a vehicle for the corporate advertising message.(p624) [24]

Much of the literature focuses on youth prevention and education programmes associated with the tobacco, fossil fuel, food and beverage industries, or corporations more generally. The activities of the tobacco industry in their efforts to develop, fund, and disseminate youth programmes are among the most well documented [5, 25–28]. Since the 1980s, the tobacco industry has developed and aggressively promoted youth smoking education and prevention programmes globally, ranging in form (e.g. school-based programmes, family self-help books, and access prevention schemes), and intended audience, including youth, parents, and retailers [5, 25–29]. Internal industry documents reveal that the purpose of these programmes was to pre-empt government regulation, including advertising restrictions, maintain access to youth, restore industry credibility and policy influence, build allegiances with policymaking and regulatory bodies, neutralise resistance from parents and educators, and ostracise public health advocates [5, 26]. Youth programmes were intended to displace those created by public health authorities and shift blame for youth smoking, emphasising the role of peer pressure and parents, while failing to acknowledge the influence of marketing, addiction, and health impacts [5, 26, 28]. These programmes were never shown to be effective in addressing youth smoking [5, 30], but the industry promoted their dissemination as evidence of their commitment to addressing the ‘problem’ of youth smoking, arguing that government-led tobacco control programmes were therefore unnecessary [5, 25, 26, 28]. In parallel, the industry actively targeted youths, including through strategic marketing [25, 31]. Beyond self-generated prevention programmes, the tobacco industry also funded and promoted externally produced programmes such as *Life Skills Training* (LST), a school-based drug prevention program recommended by the U.S. Centres for Disease Control and Prevention [28]. The promotion of LST aided commercial goals because it focused on topics such as responsible decision-making, peer pressure, and self-esteem, and, except for a single unit on advertising, avoided mentioning the tobacco industry’s behaviour as an important contributor to youth smoking [28, 29]. The tobacco industry continues to sponsor youth prevention programmes, such as *Right Decisions Right Now*, currently supported by Reynolds America Inc [32].

The fossil fuel industry similarly has a long history of providing and funding educational programmes [22, 23]. For example, BP and Shell sponsored a US-based national education programme in which children were informed that ‘it’s too soon to tell if the earth is heating up, but “a little warming might be a good thing”‘ [23]. However, fossil fuel industry influence in education extends well beyond the delivery of misinformation that fuels climate denialism and teaches children of the inherent benefits of fossil fuels; it promotes values and belief systems that align with corporate interests, and the industry has embedded itself within education policy-making [22]. Eaton and Day conclude that these:

pedagogical practices promote student subjectivities consistent with neoliberal environmentalism centred on individual actions that insulate fossil fuel industries from criticism and attempt to dissuade young people from questioning or understanding the role of corporate power in the climate crisis.(p458) [12]

Several other industries and their affiliates, including food, sugar-sweetened beverage, firearms, plastics, gambling and pharmaceuticals, produce or fund school-based education programmes that teach children about the importance of their individual choices and responsibility, and influences such as peer pressure. Industry-funded materials adopt corporate-friendly discourses that articulate narrow problem definitions and conceptualisations of harm, and conceal the role of industry and others in shaping the problem, and the industries’ *own* obligations to act responsibly and ethically. This serves to maintain discourses favourable to their positions, and shape the knowledge and values of potential future consumers [5, 6, 11, 22]. They promote neoliberal logics of consumerism, individual responsibility, freedom of choice, and the virtues of technological and market-based solutions, serving to maintain political and economic ideologies favourable to the corporate agenda [18, 22].

Despite well-documented accounts of the activities of the alcohol industry in other areas, little attention has been directed at researching its activities in relation to schools and education programmes [4] and there is no systematic research into alcohol industry-funded youth education programmes. Yet globally, alcohol use remains a leading cause of morbidity and mortality. There is no safe level of consumption and risk of harm rises with increasing level of use [33]. There is international consensus that reducing harmful use of alcohol is a public health priority, symbolised by the adoption of resolution WHA63.13 by WHO Member States, approving the 2010 *Global strategy to reduce the harmful use of alcohol* [34]. This is further emphasised by the mandate given to WHO to develop an action plan (2022–2030) to support effective implementation of the strategy [35]. WHO recommends the adoption of “best-buys” for addressing alcohol harms, including taxation and pricing mechanisms, and regulating and restricting availability and marketing (particularly to young people) across multiple platforms, and asserts the need to build support for effective policy interventions [36]. Evidently, it is critical that barriers to progress in reducing alcohol harms, including ongoing influence by the alcohol industry, are researched and addressed. In this article we define the alcohol industry as comprising major multi and transnational corporations who produce, promote and market alcoholic beverages, such as Anheuser-Busch InBev, Heineken Holding N.V., and Diageo, as well as their trade and social responsibility organisations. We adopt a narrower definition from that previously described in the literature [37], given our focus on those entities within the industry that we have identified as being active in the funding of, and engagement with, youth education programmes.

Considering what is known about the nature of youth programmes funded and promoted by other industries, and concerns about alcohol industry-funded public-facing health materials [38–43], there is a strong case for critically analysing alcohol industry-funded school-based education programmes. This study serves to address this important gap in the literature. Using a discourse theoretical approach, it aims to analyse school-based programmes provided by organisations who receive or have received alcohol industry-funding. We focus specifically on understanding what materials are being disseminated to schools by these organisations, how these materials may serve industry interests, and the various ways in which this is achieved. The analysis explores the different discourses constituted in the materials and their alignment with documented industry strategies, systematically examining the use and reproduction of industry arguments, concepts, and ideas, including individual responsibility, moderate consumption, peer pressure, simplification of complex public health issues, and deflection away from industry practices.

## Discourse theory

Discourse analysis is a widely adopted methodology for the analysis of official and corporate materials [44, 45], including those intended for educational purposes [46, 47]. This article draws on poststructural discourse theory (PDT) as established by Laclau and Mouffe and elaborated by Glynos, Howarth and others [48–50]. Following Foucault, PDT sees all social meanings and identities to be dependent on, and emerging from, historically specific systems of meaning known as discourses [51]. From this perspective, the concept of discourse encompasses not just language but a wider set of social practices and institutions which have a structuring effect on the social order which pertains at a given place and time. As such, PDT contrasts with a more limited, ideational definition of discourse as text, words and/or beliefs that are epiphenomenal (i.e. reflect an underlying objective ‘reality’) as opposed to constitutive of the social order [44, 45]. From an ontological perspective PDT assumes that social orders are radically contingent, that is to say that multiple articulations of, and ways of organising, the social world are possible and that existing societal formations are neither essential or necessary but are established and continually reproduced through discursive struggles and rhetorical practices. The hegemonic social order is always open to the possibility of re-articulation and change no matter how deeply sedimented, natural or ‘taken-for-granted’ it may appear to be [48, 52]. Established social systems are therefore inherently political [49] and involve the exercise of power, through the foregrounding of certain possibilities and meanings and the exclusion and concealment of others in the structuring of social relations [49]. Given their contingency, discourses are inherently precarious and vulnerable to the political forces excluded from or marginalised within the prevailing social order and to the dislocatory effects of unanticipated or uncontrollable events [49]. Discourse analysis within the tradition of PDT seeks to examine, describe, explain and critique the construction, reproduction and transformation of dominant discursive formations and the social meanings, practices and regimes which emerge from these [49].

Remling [45] has demonstrated the value of a combined analysis whereby the ontological and theoretical elements of PDT can be empirically operationalised through the use of analytical tools established by critical discourse analysis (CDA) as described by Fairclough [53, 54]. CDA combines three elements or stages: (1) a normative critique of discourse, that (2) enables explanatory critique of its position within, and how it contributes to, existing social orders, and (3) thereby opening a “space for challenge” and serving as a basis for action to change specific aspects of the existing order of things [54]. Building on this, Fairclough elaborates an analytical framework for the empirical analysis of texts focusing on three dimensions: *text* (formal features of a text), *discursive practice* (processes of production and reproduction, circulation and interpretation of texts), and *social practice* (implications of discursive practices for wider social practices and orders), and the interplay between these dimensions. We draw from Phillips and Jørgensen who conceptualise critique as “a positioned opening for discussion”, a resurfacing of what is otherwise taken-for-granted, exposing other meanings and possibilities, and recognising our position as being *within* discourse [52]. We adopt a retroductive approach to our enquiry, focusing on providing an explanation of a contextualised and problematised phenomenon [48].

## Methods

This analysis focuses on youth education programmes provided by organisations in receipt of alcohol industry-funding: (1) the Smashed Project [55], (2) Talk About Alcohol [56], and (3) Drinkaware for Education [57]. These programmes where chosen based on (1) receipt of alcohol industry-funding by the organisation delivering the initiative, (2) their promotion by the alcohol industry (e.g. through industry websites and corporate social responsibility (CSR) reports), (3) extent of dissemination and level of student exposure, and (4) in the case of Drinkaware and the Smashed Project, documented concerns about the misleading and biased nature of their practices and materials [4, 39, 40, 43, 58]. Teaching materials (teachers guidance notes, lesson plans, PowerPoint presentations, fact and worksheets) were obtained from teachers and websites between 2017 and 2019 (Table 1). Smashed teacher materials were obtained for both the UK and New Zealand-based version of the programme. The most recent versions that could be accessed were used for the analysis.

**Table 1:** Summary of the programmes, their provider organisations, types of materials produced.

Organisation	Programme name	Description of materials analysed	Lesson/section titles and themes
Alcohol Education Trust	Talk About Alcohol	*Teachers Manual and Guidance*	*Assessing knowledge—How much do you know about alcohol*?
		*Delaying the onset of drinking and reducing alcohol-related harm by building resilience and life skills for 11 to 18 year-olds*	*Units and guidelines–Responsible drinking*
			*Alcohol and its effects (physical and social)*
		6^th^ edition, March 2018	*Alcohol and the law*
			*Staying safe–Avoiding risk taking*
		116 pages	*Resources suitable for older students (16+)*
		9 topic-specific sections	*Top up sessions*, *myth busters and quiz*
		Supporting online activities (**Online Learning Zone talkaboutalcohol.com)**	*Facts*, *figures and commonly asked questions*
			*Involving parents*

Collingwood Learning Solutions	Smashed Project	*Teachers materials Responsible Drinking Education Programme*, 2015	*What do we know*?
			*What influences us*?
			*What could happen to me*?
		4 lesson plans	*What do I want to achieve*?*/Who can help*?
		19 pages	
Drinkaware	Drinkaware for Education	Free primary and secondary school materials:	*Introducing alcohol*
		5 lesson plans (primary and secondary versions) covering 5 topic areas	*Alcohol and emotional health*
			*Alcohol and handling peer pressure*
		Additional supportive materials • Primers • Scenario sheets • Information sheets and infographics • Worksheets • PowerPoint presentations • Teachers notes • Curriculum links • Films **Note:** each lesson plan is accompanied by all or some of the above supportive materials	*Understanding the risks and arms associated with alcohol*
			*Alcohol booster lesson*

While other alcohol industry-funded programmes are likely to exist, it is very difficult to determine the total number of such programmes and their global reach given the limited governance of, and research on, industry-funded youth education campaigns. Additionally, their content and funding streams are unlikely to be reported in uniform and transparent ways. Attempts by industries to exploit schools for commercial interests remain under-researched. This is particularly the case regarding efforts by private actors engaged in the provision of materials or other educational activities to promote certain ideas, values, and viewpoints amenable to their commercial interests but that differ in form from overt product advertising and branding activities [59]. Analysis of these three alcohol industry-funded programmes provides instructive case studies of programmes which attract alcohol industry funding and engagement, the types of resources and materials this gives rise to, and the discourses they constitute and reproduce about alcohol harms and solutions.

### (1) The smashed project

The Smashed Project is a theatre-based educational programme accompanied by teaching (background material directed at teachers and four lesson plans) and parent-facing resources. The dedicated website states that “[t]he Smashed Project is a global educational theatre programme dedicated to reducing underage alcohol consumption” and that “Smashed is now a firm fixture in over 23 countries around the world” [55]. It has been sponsored since 2005 by Diageo, international drinks company and maker of Guinness, Smirnoff, Captain Morgan and other products. The Diageo website states that the 2018 tour aimed to visit 16 countries, with Smashed appearing in 90 UK schools [60]. In an online video, the then Diageo Managing Director said “our goal is that by 2025 we will have reached over five million people across the world and educated them on the dangers of underage drinking” [61]. The Smashed Project website, which does not disclose funding sources, states that:

For participating schools, the Smashed Project is rapidly becoming a key fixture in the timetable as part of personal, social, and health studies. Teachers can follow up by utilising our teaching resources that provide a host of additional activities and lesson plans based around the story and characters of Smashed. Dedicated guides for parents and guardians can be sent home so young people are being supported at home and school on this vital social issue [55].

### (2) Talk about alcohol

Talk About Alcohol [56, 62], an education resource comprised of a teacher manual and guidance with nine topic-specific sections and lesson plans, complementary websites talkaboutalcohol.com and alcoholeducationtrust.org, and a guide directed at teenagers, was created by the Alcohol Education Trust (AET). AET is a UK-based charity which describes itself as:

…a leading early intervention charity that supports young people aged 11–25 in making more informed life choices through the 4,500 schools and youth organisations we support free of charge with our award-winning resources and training [63].

Describing the reach and impact of AET’s activities, the AET website states that:

Allowing 1 year group per school, we estimate that over 700,000 children in over 4500 settings used Talk About Alcohol games, films, lesson plans. This includes 54 new schools in Scotland, 63 new settings in the Midlands and a further 162 across the South and west during the 2019/20 academic year [64].

The Founder and Director of AET is also Executive Director of “Alcohol-in-Moderation” (AIM), a journal and database of alcohol research. She is also Co-Director of The International Scientific Forum on Alcohol Research (ISFAR), which provides commentaries on newly published research papers [65]. A 2014 editorial in the journal *Addiction* identified what it called “a convoluted set of financial connections” among ISFAR, AIM, Boston University’s Institute on Lifestyle and Health, and the alcohol industry, the latter providing funding via subscriptions and project grants [66]. However, this was rejected by AIM’s Executive Director [67]. ISFAR’s extensive conflicts of interest in relation to alcohol have also been described elsewhere [66, 68]. Recent AET funding includes the Distillers’ Charity (the charity of the Worshipful Company of Distillers), and the Wine and Spirit Education Trust (funded by Diageo, Chivas Brothers/Pernod Ricard, among others) [69–71]. Further funding has been received from LEAF (Lifeskills Education and Alcohol Foundation), a charity established under the UK Responsibility Deal, with funding from the Portman Group (a CSR body representing major alcohol producers), Heineken and others, and administered through a blind trust [72, 73]. According to recent AET Trustees’ Annual Reports, 40% of its funding in 2017/18, 22% in 2018/19, and 21% in 2019/20 came from corporate partnerships [74]. AET entered a partnership in 2013 with ABInbev’s “Family Talk About Drinking UK”, an initiative launched in 2011 [75].

### (3) Drinkaware for education

Drinkaware, an alcohol industry-funded UK-based charity, produces educational resources for teachers and parents, among other materials and programmes. The schools-based materials include guidance notes, lesson plans, fact and worksheets, films, and PowerPoint presentations. The website states that:

Drinkaware is an independent charity which aims to get people to think differently about alcohol. Our entire focus is on getting people to understand the harm it can do to their health, families and those around them. If people understand the impact drink can have, they’re more likely to make a change. Established in 2007, Drinkaware works alongside the medical profession, the alcohol industry and government to achieve its goals [76].

Drinkaware currently produces a range of “curriculum-linked education resources aimed at teaching nine to 14-year-olds about the harms and risks associated with alcohol”, collectively referred to as “Drinkaware for Education” directed at educators in the UK [57, 76]. All materials are freely available through the website, accompanied by other items including a booklet directed at parents titled “Talking to your kids about alcohol” [57]. Previous analyses of Drinkaware’s materials directed at adults have found that some content normalizes alcohol use and provides cues to alcohol consumption [40, 43]. Drinkaware’s website and social media account have also been found to contain misinformation and selective omissions relating to alcohol harms, including information on cancer and alcohol use during pregnancy [38, 40]. Furthermore, recent concerns have been raised by an independent public health advocacy organisation, Alcohol Action Ireland, about the access Drinkaware Ireland (a related organisation with similar funding arrangements and concerns raised in the literature [40]) has to schools and the associated conflicts of interests [77]. The Drinkaware for Education webpage or Drinkaware’s main website do not appear to report on the reach or dissemination of their educational resources, and we could not locate a formal evaluation of their use or impact.

### Data analysis

We analysed teacher and youth-facing materials provided through the Smashed Project, Talk About Alcohol, and Drinkaware youth alcohol prevention programmes, including guidance materials directed at teachers, lesson plans, fact and worksheets, PowerPoint presentations, and linked on-line materials (Table 1).

**Fig 1:**
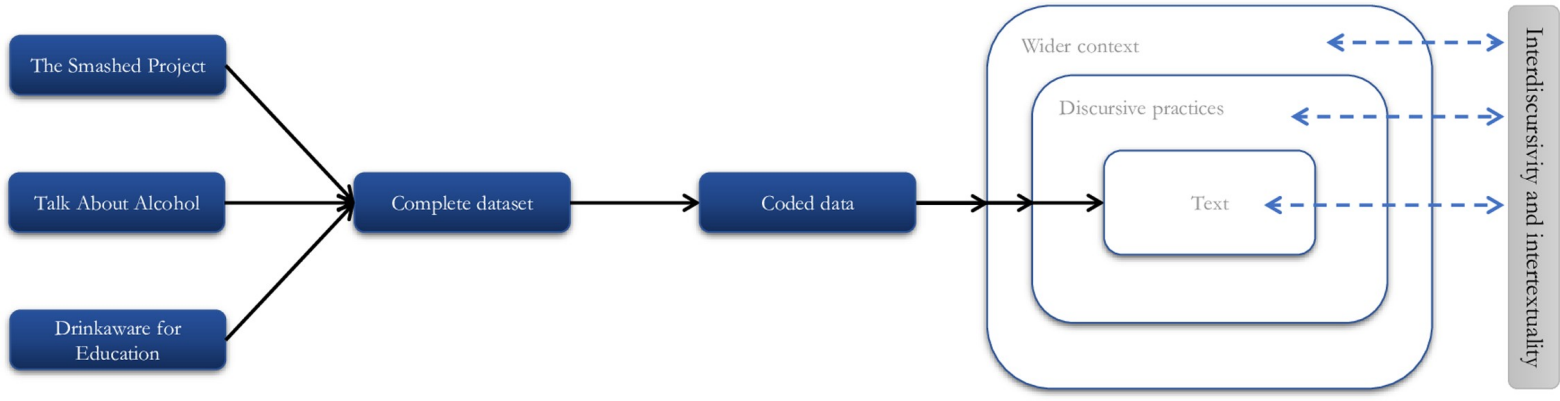
Schematic representation of the analytical process.

Two researchers from the field of the commercial determinants of health with experience in qualitative document analysis (MvS, MP) independently read all material in-depth to become familiarised with the content. Initial coding frames were constructed independently by each researcher, based on close reading of the data. Images and figures were analysed as constituting discourse by integration through their meaning as determined by their deployment within the documents [54]. Coding was informed by previous literature on industry-funded youth education materials, focusing on themes related to problem definitions, solutions, roles and responsibilities, harms and influences. Coding was ‘open’ in that it was guided by these conceptual codes but complemented by the identification of unexpected or emergent codes as the analysis proceeded [45]. An iterative approach was adopted, whereby all relevant data were identified through constant comparison, meaning each unit of data was compared with the remainder of the data to establish a comprehensive body of analytical elements which were used to construct a coding frame. Coding was supported by the use of Microsoft Excel, facilitating the recording of emergent themes and associated textual elements. Consensus on this initial coding frame was then established through open discussion. This coding frame was then used to reanalyse the entire dataset, with additional analytical elements emerging upon re-reading of the material, which were then integrated into the final coding frame again through discussion to reach consensus. Guided by Fairclough’s three-dimensional model and open discussion between the researchers, the coded data was analysed to identify formal features of the text (e.g. use of language, evidence, and arguments), the discursive practices, relation to wider social practices, and the interplay between these elements [44, 52, 54]. At this stage we also drew upon the concepts of interdiscursivity and intertextuality, capturing the ways in which different discourses are articulated together, and prior discourses and texts are drawn from (i.e. capturing the historical influences on texts), respectively (Fig 1) [44, 52].

## Results

Our analysis revealed that these programmes serve to reproduce hegemonic industry-favourable discourses of personal responsibility, moderate consumption, and individualised problem definitions and interventions, linked to a concealing of the role of industry practices as drivers of harm and inequities. This was evident in the way various discursive practices and textual features were brought together within the material to form an overarching discourse centred on the concepts of ‘responsible drinking’ and ‘the good consumer’ and problematising of youth. The good consumer adopts the ethos of responsible drinking by: not partaking in underage drinking; being in control, managing stress, and able to resist peer pressure; being familiar with the product including physical properties as well as harms and benefits; and learning the rules that guide ‘responsible’ adult alcohol use. Within this discourse, the problem is therefore portrayed as arising when individuals do not adhere to these practices and norms. The aim of such programmes is therefore captured within a limited discourse of individual skills and knowledge building, including developing personal coping skills, and awareness of one’s personal obligations to be in control and act ‘responsibly’. In this way the materials introduce a moralising aspect to teaching about alcohol, and create a conceptual vocabulary that precludes articulating a structural and context-dependent account of alcohol harms and solutions that involve collective action. We provide a more detailed analysis of the discourses drawn upon in these materials, focusing on the ideas, conceptualisations and taken-for-granted ‘truths’ within the text, followed by a discussion of how these texts serve to maintain social practices and public understandings favourable to corporate interests and maintain corporate power in their ability to shape discourses and value systems. Unless the specific programme and/or type of material is explicitly stated in the preceding text, example quotes presented are followed in brackets by the name of the relevant programme to guide the reader to the quote’s source.

## Discourse of self-control and personal responsibility

Underage drinking is a central topic within the materials, and although problematised as a concerning social issue in need of intervention, articulation of the breadth and extent of alcohol related harms, their impacts (both direct and indirect) and the complex nature of these issues were minimised by the discourses. Instead a multifaceted major public health issue is distilled down to a limited number of determinants of young people’s drinking, such as the family environment, peer pressure, poor coping mechanisms, or misuse of alcohol; “[y]oung people may start to drink with the consent of their parents as part of family life, or they may choose to drink secretively due to peer pressure” (Smashed Project Teaching Materials).

Teachers and students are alerted to the impacts of underage drinking:

Common problems young people experience are the effects of severe intoxication and accidents. Studies suggest that young people combine alcohol and sex, especially prior to their first sexual experience and that there is a link between drinking before sexual activity and unsafe sex (Smashed Project Teaching Materials).

However, the importance of the issue is minimised by the assertion that only a minority of individuals will go on to experience significant harms: “[a]lthough young people may at times drink in an uncontrolled way, most will not go on to develop serious problems” (Smashed Project Teaching Materials). This underplays the serious impacts on some young people; those who die by suicide, or injuries, which are often related to alcohol consumption [78]. This framing also conceals the wider social and economic impacts of such events, and longer-term effects of moderate drinking. The impacts and implications were similarly lacking in detail and emphasis in Drinkaware and Talk About Alcohol materials.

The discourse used within the materials from all three organisations serves to maintain the view that it is young people, their uninformed and bad choices and behaviours, their culture, their lack of resistance to the influence of others (particularly peers) and inability to stay in control or manage stress, that constitute the problem, while alcohol itself, when used responsibly, is a normal, if not beneficial, product that adults enjoy. Building on this line of argument, it is therefore the moral responsibility of individuals as ‘good citizens’ to know what influences drinking behaviours, what constitutes ‘responsible’ choices, and to learn how to resist external pressures. These concepts appear to inform the aims, purpose and content of the programmes. For example, the Smashed teaching materials aim to; “[e]xplore peer pressure including the role of the media and social media”; “[r]aise awareness of personal responsibility in making decisions about alcohol”; “[t]o identify how other people can influence our behaviour” and:

This simple and fun exercise will help students clearly identify with the power of peer pressure and how it can make us feel. Peer pressure is the most significant influence on young people’s behaviours outside of the home. It is argued that it is in fact more powerful than parental influence entirely (in terms of teenage development).

Drinkaware materials state that they will teach children “[t]o understand how young people’s actions in relation to drinking alcohol can be influenced by their peers” and “[t]o understand that peer pressure can lead to underage and irresponsible drinking” (*Alcohol and handling peer pressure*, secondary level lesson plan). In one lesson plan titled *Alcohol and handling peer pressure* (both primary and secondary level) teachers are instructed that “[i]f it hasn’t come up in discussion already, explain to the class that peer pressure can lead to young people drinking underage.” The Talk About Alcohol Teacher Manual and Guidance also focusses on peer pressure and personal responsibility:

We also aim to reduce the prevalence of ‘drinking to get drunk’, and to encourage self reliance [sic], confidence and self respect [sic], making it easier for young people to resist peer pressure.

The focus is on encouraging young people to take personal responsibility and to choose behaviours that resist social coercion and peer pressure.

Parents and siblings are also cited as key influences, and, though rarely, the media or social media (but not the industry’s use of these channels); “[s]tudents explore feelings and influences (including social media) involved in peer pressure” (Lesson Plan 2, *What influences us*?, Smashed Project Teaching Materials). However, peer pressure receives the greatest focus and is the most heavily emphasised causal influence with building resilience to peer pressure and responsible decision-making being presented as the logical solutions, which the materials can provide.

The discourse is equally powerful in what is not articulated, and thereby concealed in the process. Alcohol advertising, accessibility, availability or pricing, all of which influence youth and adult drinking behaviours, and the alcohol industry’s influence over these aspects of alcohol regulation, are not included in the problematising of underage drinking. This omission is evident in lesson plans intended to teach students about what influences behaviour. For example, according to lesson 2 of Smashed teaching materials, *What influences us*?:

The teacher then encourages broad thinking about what influences us as individuals–the weather, the news, an argument, friends, Facebook etc. Pupils should put into rank their order of importance. Discussion. Why do things influence us? Why and how are we influenced by Social Media? What is the word for when friends influence us?

As noted above, Smashed materials claim that, outside the home, peer pressure is the most significant influence. There is no elaboration as to *why* young people may encourage their peers to consume alcohol. At no point are teachers and students encouraged to reflect on the role of society and government in acting to protect children and young people from the harms of alcohol and targeting by industry, on the goals, nature and effects of advertising, or of the industry’s obligation to act ethically.

The apparent broader ‘goal’ expressed within the materials, beyond identifying influences of underage and irresponsible drinking, was to produce responsible youth who are in control, take personal responsibility for their choices and actions, and who value these virtues:

Research shows that if alcohol education through school is going to have an impact, the message that responsible drinking doesn’t have to mean having less of a good time must be key. Rather, education messages should emphasise that you are more likely to have a good time if you are in control…Many of the life skill elements of the lesson plans and worksheets cover issues that are relevant to risk taking and the importance of taking personal responsibility in general (Talk About Alcohol Teacher Manual and Guidance).

Similarly, “[t]he Smashed Project is dedicated to breaking the culture of underage drinking around the world. Through creative education, we can equip young people with the information, awareness, and confidence to make responsible choices around alcohol”(Smashed Project Teaching Materials).

Accepting individual responsibility is partnered with encouraging discussion about responsible behaviours and identifying individual-level solutions; “The session gives students the opportunity to reflect on their knowledge and opinion of alcohol and to begin to discuss what responsible behaviour entails” (*Assessing knowledge–how much do you know about alcohol*?, Talk About Alcohol Teacher Manual and Guidance). Young people are to be informed about their obligation to identify and implement individualised solutions to personal issues, described by Drinkaware, for example, as “‘healthy ways of coping with stress’… in order to avoid relying on addictive substances like alcohol” (*Alcohol and emotional health*, secondary level lesson plan). Students should also be able to identify what works for them:

We can all find ways of managing problems and stress without alcohol. Distribute the **Top ten stress busters pupil sheet**. Pupils can work individually to rank them in their own order of what they think would work for them. (*Alcohol and emotional health*, primary level lesson plan, Drinkaware for Education, emphasis in original)

Partnered with this individualising discourse surrounding drinking behaviours, concepts of the amount that can be drunk and the impacts of consumption were conveyed as being fluid and variable. This ‘vague’ discourse serves to again focus on the role of the individual. In the following characterisation, decisions not to drink, can apparently include “drinking”:

## When not to drink

Responsible drinking means drinking enjoyably, sociably, and moderately and includes not drinking at all in situations when the effects of alcohol will put your own or someone else’s safety at risk, (*Staying safe–avoiding risk taking*, Talk About Alcohol Teacher Manual and Guidance, emphasis in original)

The concept of enjoyment was also articulated with the concept of drinking in moderation:

For adults, drinking in moderation (one or two drinks) is usually a pleasant and relaxing thing to do and leads to no harm most of the time. But there are times when you shouldn’t drink and these include if you work with machinery or at heights, as even small amounts of alcohol affect coordination, concentration, reactions and judgement (*Test your knowledge* online quiz, Talk About Alcohol).

The phrase “responsible drinking” is a strategically ambiguous term which is used almost exclusively by industry or industry-funded organisations [39]. Such framings place a strong emphasis on individual responsibility and staying in control. The phrase “responsible drinking” appears 28 times in the 116-page Talk About Alcohol Teachers Manual and Guidance. This phrasing narrows the conceptualisation of the term ‘safe’, by implying that alcohol use is only a risk to people’s ‘safety’ in specific situations. However, this underplays the evidence that the vast majority of alcohol harms occur through longer-term consumption, irrespective of situation.

## Discourses of consumption: Product ‘information’, familiarisation, and normalisation

One important way to produce responsible consumers is to shape what they know about alcohol, how they value its consumption, and how harms and benefits are conceptualised. This knowledge-shaping is achieved by these educational materials through the provision of an array of product ‘information’. The discourse includes positive framings of the product, familiarising schoolchildren with its production and use, and normalising its use in society as well as presenting a range of social and economic impacts.

### Information, familiarisation, and normalisation

Students are presented with information through lessons which frame alcohol as a ‘natural’ product which is socially and economically beneficial. It includes teaching them how alcohol is made, with an emphasis on ‘natural’ products and processes, as in this detailed description of brewing and distillation in a Talk About Alcohol question and answer-based lesson: “[i]n cider making, crushed apples are used. Beer is produced from malted grain—usually barley—to which hops have been added for flavour.” Similar content features in a Drinkaware lesson plan for “[i]ntroducing” primary school pupils to alcohol:

To make alcohol, you need to put grains, fruits or vegetables through a process called fermentation (when yeast or bacteria react with the sugars in food–the by-products are ethanol and carbon dioxide).

Wine and cider are made by fermenting fruit, while beer is made from fermented cereals such as barley and rye. Spirits can be made from fermented fruit or cereals (*Introducing alcohol*, primary level lesson plan)

Again, the discourse of production is selective. For example, there is no mention of other potential constituents such as added sugar, preservatives, flavourings, refining agents, or nitrogen. The environmental and human impacts of alcohol production, transport, and sale are similarly absent [79, 80].

Information on the positive role of alcohol is used to shape children’s attitudes to alcohol, in this case as a natural way to relax or alleviate stress:

Question 3: Why do many adults choose to drink?

Answers may include: to relax, be sociable, unwind, forget worries, stress, to feel more confident.

Question 4: Where do people choose to drink?

At this point you can use the risk continuum, whereby you ask children to rank the reasons for

drinking and places where people might drink as high risk, medium risk or low risk. Hence drinking in the park might be chosen as ‘high risk’, whereas drinking as an adult to relax might be categorised as ‘low risk’.

[…]

Stress? A little alcohol will help you feel relaxed and unwind, but more than that and it has a depressing effect (*Assessing knowledge–how much do you know about alcohol*? Talk About Alcohol Teacher Manual and Guidance).

Similar messages are deployed in Drinkaware’s secondary school lesson plan (*Introducing alcohol*), which includes the advice “[i]n small doses alcohol can make you relaxed and happy, but too much of it can increase anxiety and stress, rather than reduce it”. This information is to be provided to students by the teacher alongside a presentation slide with the statement “[d]rinking alcohol makes you happy” and a marketing-type image of young adults smiling, having a meal together, and drinking what appears to be wine (*Introducing alcohol*, secondary level presentation). The deployment of similar positive framings and mixed messages is also commonly identified in alcohol industry CSR materials directed at adults [43].

The normalisation and familiarisation of alcohol consumption is exemplified in one Talk About Alcohol lesson by an exercise (*games—musical chairs games–how we are influenced by alcohol*) in which younger children learn about types of alcohol, including brand names: “You can name five brands of alcohol (Bacardi, Smirnoff, Carlsberg, Stella, Blossom Hill, etc.).” Familiarisation is further facilitated by a focus on alcohol units and laws governing alcohol use:

The Alcohol Education Trust aims to work towards a more responsible drinking culture in the UK, by ensuring young people are able to make informed choices based on knowledge about units and guidelines, alcohol and the law, alcohol and its effects, how to stay safe and how to resist peer pressure (Talk About Alcohol Teacher Manual and Guidance).

The Talk About Alcohol materials also teach children about the economic benefits of alcohol consumption, placing the health impacts of alcohol consumption in context:

Objectives: To consider the contribution of a product to a market economy from different angles, including the effects on individuals as well as the wider community (*Alcohol and the community*, Section 3).

This section of the manual directs teachers to a worksheet for 14–16 year olds that includes an activity called *Balancing Act*, which begins: “Now it’s time to consider the ways in which alcohol and social drinking can make a positive and/or negative contribution to your local community and to individuals”. In this exercise students are advised to design a presentation and given a list of things to consider in the “balancing act”. One health harm is mentioned: ‘alcoholism’, and one explicit social harm: ‘fights’. To “balance” this selective list of harms, students are prompted to consider the positive impacts of alcohol including the impacts on jobs, drinkers, bars, “lively city centres”, the “[d]rinks industry”, and on glass and bottle manufacturers. This lesson draws from neoliberal rationalities about the importance of ‘the economy’ and the balancing of different considerations based on cost-benefit analysis, foreclosing discussion on the possibility of prioritising health, or social justice over economic considerations. In parallel it overlooks the inequitable distribution of the harms and benefits.

### Selective presentation and omissions

Industry discourses are strategic in nature with regards to what is articulated and practiced, how ideas, frames and evidence are used, as well as what is *not* articulated and therefore concealed. Alcohol industry-funded CSR organisations have been shown to disseminate misleading information about health harms. Cancer denial or misrepresentation of risk is a particular focus of alcohol industry strategies [4, 40, 42, 81], consistent with observations across other industries over many decades, from asbestos, tobacco, pesticides to pharmaceuticals and food, who have sought to conceal or deny the cancer risk posed by their products [82–85]. The risk of breast cancer is often omitted (or sometimes simply denied by industry sources), which is notable given the importance of female consumers to the alcohol market [86]. Colorectal cancer is also frequently selectively omitted. Drinkaware lessons omit mention of specific cancers from ‘information’ on long-term health risks, while including others (e.g. liver disease). For example, the primary lesson plan *Understanding the risks and harms associated with alcohol’* and accompanying YouTube film (aimed at 9–11 years), PowerPoint presentation and information sheet, do not include cancer, and the secondary level lesson lists “getting cancer” as a long-term harm.

Similarly, the Drinkaware *Alcohol and effects on the body* information sheet for secondary school includes liver disease, but omits information on the risk of cancer, instead posing a question and referring students to the Drinkaware’s main website for further information: “[h]ow does alcohol affect these parts of your body: bowel, stomach, breast cancers?” Selective omissions also occur in Talk About Alcohol materials. The online information, *Body watch* (an interactive infographic of the body), on the effects of alcohol omits mention of the risk of cardiovascular disease referring only to “irregular heartbeats”, and does not mention colorectal cancer, or breast cancer (facilitated by only depicting a male body), mentioning only liver cancer [87]. The Talk About Alcohol Teacher Manual and Guidance does contain information on the risks of cancer, including breast cancer. However, the information is misleading in that it suggests that the risk of cancer is due to excess intake, whereas the risk of bowel and breast cancer, for example, increases linearly with alcohol use. Smashed materials likewise mention only mouth and throat cancer, omitting the most common cancers, breast and colorectal (Smashed Teacher Resource, New Zealand version). According to Smashed, only heavy alcohol use (which is undefined) is harmful: “[r]egular, heavy alcohol use can damage your liver, heart, stomach and brain” (Smashed Teacher Resource, New Zealand version). There is no mention of cancer in the UK Smashed materials analysed. When cancer is mentioned, the level of risk in relation to amount of alcohol consumed is not provided. Furthermore, health risks are often listed with no grounding as to their impacts or likelihood, and little connection is made between underage drinking and longer-term harms in adulthood, instead predominantly focusing on immediate short-term impacts of a given drinking episode. There is little attempt to portray the burden imparted by such health issues on individuals, those close to them, or their communities, and most of the images used are benign in nature.

Mental health issues, self-harm, suicide, violence, death, and crime are introduced by the resources. However, mental health impacts are portrayed as being a result of people’s poor coping skills and inadequacy in dealing with stress in ways other than drinking alcohol. There is also a focus on the technical and physiological aspects of alcohol consumption–what it does to the body and how the body processes it, linking this to more immediate effects such as flushing, slurred speech, and diuresis. Addiction is similarly defined in physiological terms, that is, the processes of dependence, withdrawal, and inability to stop drinking, with little reference made to the consequences of addiction on people’s lives. For example, Drinkaware advises teachers to:

Explain to pupils that tobacco and alcohol are the most commonly used drugs in the UK and a lot of people are dependent on them. Ask them if they can name any soap operas they may have seen which involve places where people drink alcohol–and take feedback from the group. Examples might include:
  • Coronation Street–The Rovers Return
  • EastEnders–The Queen Vic and The Albert
  • Hollyoaks–The Dog (in the Pond) and The Loft nightclub
As well as drinking alcohol in pubs, bars and restaurants, people may also drink alcohol at home, for example, when eating a meal, at a party or special occasion, watching TV.
Explain that most people who drink too much alcohol don’t enjoy feeling dependent. That’s why a lot of people try to stop. It isn’t easy for people who are dependent to stop smoking or drinking alcohol on their own. They need help and support (*Alcohol and emotional health*, primary level lesson plan, Drinkaware for Education).

In another Drinkaware primary level lesson plan titled *Introducing Alcohol*, teachers are instructed to ask students to express their “opinions and thoughts on alcohol” (by standing along a continuum from strongly agree to strongly disagree) in response to certain statements (set out below) which are provided to the teacher. These are accompanied by suggested responses “to incorporate into the class discussion”. For example:

1. Drinking alcohol makes you happy
  • In small doses alcohol can make you relaxed and happy, but too much of it can increase anxiety and stress, rather than reduce it.
  • Alcohol can affect your judgement, sometimes leads to depression and has been linked to self-harm and suicide in young people.
  (Main activity, Alcohol: fact or fiction?, *Introducing Alcohol*, primary level lesson plan, Drinkaware for Education)

The Talk About Alcohol Teacher Manual and Guidance also articulates addiction as being the result of an individual’s “abuse” of alcohol and focuses on characterising the reasons why withdrawal is unpleasant or the need for increasing consumption:

The amount of alcohol in drinks can be increased by a boiling process called distillation. This makes spirits such as gin, vodka, whisky and rum, which usually contain about 40% pure alcohol. (For more information see **page 104**). Alcohol is a legal drug, a drug is defined by the UN as something you take that changes how you think feel or act. Alcohol is a depressant, in that it slows down your nervous system and alters your mood, behaviour, judgement and reactions. If abused, it can lead to dependency or addiction.
What is your general attitude towards alcohol?
Good (e.g. celebration, relaxation, sociable, etc.)
Bad (e.g. health risks, personal risks such as accidents, violence and disorder, addiction).
Think about how much, with whom and where.

**Q3 Those who persistently drink too much can become addicted to alcohol**. **Kicking the habit is exceptionally difficult. Why?**
**c) Because alcoholics** **feel wretched without alcohol**
There is alcohol tolerance and alcohol addiction.
Toleration is when you gradually need more and more alcohol to achieve the same effect.
Addiction means that you can no longer cope without alcohol. You feel you have to drink.
Without alcohol you feel sick and have withdrawal symptoms. You start trembling, shivering, feel nauseous or even have to vomit. These withdrawal symptoms make it very difficult to overcome addiction, and specialist help and support is needed (emphasis in original).

These approaches to conceptualising addiction serve to locate the issue within individuals and their ‘inappropriate’ use of the product obscuring the processes of normalisation, glamorisation, and heavy marketing of an addictive product or the role of wider social determinants as drivers of addiction and the inequitable impacts.

Alcohol industry-funded materials are also known to omit or obscure mention of the risk of pregnancy, either omitting it entirely, making it difficult to find, and/or disputing that a risk exists [38]. While the risk of unsafe sex or unplanned pregnancy is at times mentioned, Drinkaware’s lesson on “understanding” the risks and harms associated with alcohol include a range of short- and long-term harms, but omit risk of pregnancy and the risks and impacts of Foetal Alcohol Spectrum Disorders (FASD). Pregnancy and FASD are included in the Talk About Alcohol materials.

Alcohol marketing is a significant influence on the age at which young people start drinking, the amount they drink when they do start, and peer norms [88]. However, as mentioned above, a striking absence from the discourses is any mention of the role of the alcohol industry and marketing in youth drinking. The only reference we identified to advertising was in the Talk About Alcohol Teacher Manual and Guidance (section 8 *Involving Parents*), where it was suggested as part of an exercise for children to do with parents at home:

A great way to engage parents subtly is to send a task home, such as the quiz (which they can do together), or to design a poster on an aspect of alcohol, such ‘how much is too much’ (including units and guidelines) or avoiding risk taking. You can ask them to assess an alcohol advert or count how many times they see alcohol on a particular programme and what effect this may have on behaviour, product choice, acceptance etc.

No further information is given as to how to identify marketing practices within the advertisements or question their intended effects, and as it is only mentioned as an “at home” exercise, it is discretionary and may not be completed. There is also little if any mention of the impacts of alcohol outlet concentration on risks of harm at the community-level [89], and that alcohol use and the influence of the industry are recognised as major global health issues [3].

Echoing wider industry discourses, all three organisations repeatedly frame harms as arising from heavy drinking (not defined) and alcohol ‘misuse’ and ‘abuse’. The term implicitly suggests that appropriate ‘use’ is therefore not associated with the harms assigned to ‘misuse’. At the same time, it is frequently iterated that most people use alcohol to relax, and deaths are due to ‘misuse’ (e.g. Talk About Alcohol Teacher Manual and Guidance), thereby creating a dichotomy–safe, enjoyable use versus unsafe misuse, the latter confining the harms to a pattern of drinking that is undefined and nebulous. This type of misinformation is often partnered with another alcohol industry strategy that undermines messages about harm through the presentation of mixed messages, and/or marketing-style images [40, 43]. This was observed in the Drinkaware materials where statements about alcohol harms appear alongside couples clinking glasses of alcohol or joyful groups of people consuming alcohol. Of note, overall the discourses appear to prioritise teaching children and young people ‘about’ alcohol as a product, the different forms and unit measurements, how it is and can be consumed and in what contexts, harm-reduction measures such as consuming with food and designating a non-drinking driver, the legal aspects, and alcoholic beverage production and physiological effects, over teaching about the short and longer-term harms and the impacts on families and communities in a meaningful and comprehensive way.

### Shaping behaviour and choices

The materials from each of the organisations contained a discourse that appeared to promote familiarity and normalisation of alcohol as a consumer product. This is achieved, as elaborated above, through lessons presenting it as a natural product. Elsewhere, familiarity may be enhanced through describing positive behaviours, scenarios and emphasising positive statistics:

Reassuring stats… Among 16–24 year-olds in 2016, 16% of men and 17% of women said they binge drink. That means an overwhelming majority of young adults (84% of men and 83% of women) go out to enjoy themselves and socialise, not to get drunk (Talk About Alcohol Teacher Manual and Guidance).

Positive and negative drinking scenarios are presented to teachers and students in a Talk About Alcohol lesson *Just a few drinks* in which students watch 4 BBC2 video clips about how much is *too* much alcohol:

Positive group scenarios… Jordan realises after a few swigs from the bottle that the neat brandy (40% ABV) is not a good idea and so switches to drinking a beer slowly (see information on units and guidelines via: alcoholeducationtrust.org/teacher-area/units-and-guidelines or page 21).

The message is that drinking in young people is normal, and that problems occur when “too” much alcohol is consumed. The discourse is centred on the logic of ‘getting the balance right’ by being a good and responsible consumer of alcohol. Consuming alcohol in a ‘balanced’ way is sometimes portrayed as equivalent to not drinking at all–no or two drinks are all ‘balanced’ forms of use–which was portrayed pictorially in one Talk About Alcohol activity titled *How much is too much*? *Getting the balance right*. Using a seesaw image, the “right” balance is represented by zero to two drinks; maybe even three, as this option is absent from the image. Getting the balance “wrong” evidently only applies to 4 or more drinks (units are unspecified in this activity). Children are instructed to: “[d]raw lines between the number of drinks and their likely effects?”. The image articulates an equivalence between harms. For example “have slurred speech”, “get in trouble with the police”, “say things you will regret” and “get injured” are all floating in the image and waiting to be assigned to the corresponding number of drinks. The exercise thereby forecloses the opportunity to consider and debate the considerable differences regarding the impacts of each “effect” on individuals and society. Such descriptors also draw on stereotypical and moralising assumptions about youth, their behaviours, and experience with alcohol use, again placing the locus of the problem within youth, poor choices and lack of control.

Teaching how to get the balance right also appears to involve ‘teaching’ children how to drink to obtain enjoyment, but without ‘losing control’. It has been shown that alcohol industry CSR campaigns tend to focus on visible ‘out of control’ behaviours (e.g. drink driving, underage drinking) which pose a risk to their corporate image, while using vague or undefined concepts like “moderation”, “control” and “responsibility”, thereby avoiding being too restrictive by imposing specific drink limits [39, 40, 90]. The consumer is intended to judge for themselves what is the ‘right’ amount for them, up to the point at which they ‘lose control’. This industry discourse, with an emphasis on “control” and nebulous individualised amounts that achieve this control is also adopted in these materials, as in the Talk About Alcohol Teacher Manual and Guidance:

How much is too much? Getting the balance right - …if you spot the signs in yourself or a friend, moderate your or their drinking or stop drinking further amounts. The last thing you would want is to lose control, vomit or end up in hospital.

… the message that responsible drinking doesn’t have to mean having less of a good time must be key. Rather, education messages should emphasise that you are more likely to have a good time if you are in control.

Drinking in an ‘uncontrolled’ way also appears in the Smashed materials. In another Talk About Alcohol lesson, suggestions for “[a] good party” appears to include prompts to consider alcohol:

Suggested examples:
**A** **good party**
Nice venue, preferably in a big flat or house.
Just the right amount of people.
Good atmosphere and music.
Good food and drink (alcohol?)…
Meet up and get ready with friends first to get in the right mood (with alcohol?).
Have alcohol, but without getting drunk or throwing up (emphasis in original).

Some Talk About Alcohol lessons appear to be about teaching schoolchildren specific drinking skills. Their *Units and guidelines*: *Responsible Drinking* lesson for 14–16 year olds advises on the importance of reading the label. Drinkaware’s *Alcohol Booster session* for secondary schools, as well as including misinformation about health harms, involves lessons in pouring drinks:

Next, using the biggest wine glass you have, ask one student to imagine they are an adult pouring a glass of wine at a party. Where would they fill it up to? Then pour this into the unit measure. This will show the class/group how easy it is to pour more than a standard glass of wine.

## Discussion

Analysis of three alcohol industry-supported school-based youth prevention programmes revealed many commonalities. All employed discourses that serve to maintain and reproduce wider hegemonic social logics of individual responsibility, moderate consumption, and narrow problem definitions and causes, thereby aligning with corporate interests. The discourses employed articulate underage drinking as a problem located within youth themselves: youth inherently make poor and uninformed choices, are deficient in skills to resist peer pressure, and lack control. The logical answer is then portrayed as addressing these deficits through the provision of information about harms, the powerful influence of peers, and a consumer’s moral obligation to use information, coping skills, and self-restraint to stay in control and act responsibly when consuming alcohol. These conceptualisations conceal the responsibility and duty of other actors (e.g. industry and governments), and the possibility of collective action to promote health and prevent harm.

All programmes promote familiarisation and normalisation of alcohol as a ‘normal’ and enjoyable consumer product which children must learn about and master how to use responsibly, which is achieved by teaching about alcohol production, units, social benefit, ‘the law’, ‘responsible’ decision-making, managing stress, and finding the right ‘balance’ for them as individuals. The role of the industry, and marketing practices are near-invisible across the three organisations’ materials. Absent from the discourse is any mention of the impacts of price, availability and access, or the impact of alcohol use on poverty, and inequities, demonstrating concerning divergences from public health discourses which problematise the product and lack of population-level policy interventions. Similar to tobacco industry-produced materials, there is a focus on ‘the law’, and specific health harms were absent from some lessons. While a number of harms are presented, the ways in which they are articulated (both in textual and image form) leaves little ability to apprehend the risk of specific outcomes or what such harms *mean* for an individual, those close to them, or their communities, and concepts like addiction are presented in limited ways. Harms are predominantly framed as having impacts at the individual level, and often described in technical terms and physiological impacts (e.g. dehydration, diuresis, physiological dependence, and symptoms of withdrawal). The discourses constructed in these materials align closely with those of other alcohol industry CSR discourses which employ selective presentation of harms (particularly cancer), and ambiguous terms such as “responsible drinking” [39, 40, 42]. This is particularly concerning given evidence that young people cite health considerations as important influences of their decisions relating to alcohol use [91].

These discourses serve to centre the problem on the individual and their ‘choices’, and clearly try to navigate the challenge of appearing to address the harms associated with alcohol use while promoting consumption and avoiding being too prescriptive about what constitutes a safe level of consumption–this is for the individual to decide [39]. Moderation is promoted as the socially desirable and acceptable drinking pattern that youth and adults *should* aspire to adopt. As elaborated by Room, the promotion of personable responsibility and moderation (often undefined) as methods of controlling alcohol use serve as solutions to a core contradiction in neoliberal consumerist societies that simultaneously assign moral virtue to self-control while endorsing free market ideologies and marketing activities that stimulate increasing consumption [92]. It is the individual’s moral obligation to exercise their freedom and rights as a citizen through consumption while aspiring to master self-restraint and control. According to this logic, when harm does occur, it is the fault of the individual, their lack of will power or adherence with the guidelines, the consequences of which are for the individual, not society or government, to take responsibility for. The materials analysed constitute, and serve to reproduce, this wider ideological project, that is not limited to alcohol and health but pertains to the wider consumerist economy which tends to favour corporate interests.

While the materials include content that conveys the economic arguments for the importance of alcohol consumption to the night-time economy, and bottle manufacturers, no opportunity is provided to teaching how to understand, and critique these arguments. No space is dedicated to teaching about collective action, and advocacy for policy change, or the democratic rights of citizens and communities to decide how, when and where alcohol should be available to whom and at what price. The materials do not cover the growth in, and role of, corporate influence, particularly in relation to advances in strategic marketing practices, and the right of teachers and students to challenge the legitimisation and normalisation of these practices. Essentially, alcohol harms, including the devastating impacts of underage drinking, representing major global health issues with complex social, commercial, and political determinants, are decontextualised, depoliticised and distilled down to the individual child who must make the ‘right’ choices and become responsible future consumers. It is not our intention to suggest that the materials contain no factual content or that youth should not be informed about the impacts of their choices, peer pressure, or the harms associated with specific products, or to question the importance of addressing underage drinking and strengthening youth decision-making. We seek to problematise the highly misleading manner in which the problems, harms, causes, and solutions are articulated and conceptualised, and to show what is concealed in the process.

As described by Jackson and Dixon in relation to Smashed [4], these materials give a veneer of being suitable for educational purposes by, for example, referring to harms, peer pressure and decision-making, which are legitimate concepts in the context of substance abuse prevention. However, the dominant focus on peer-pressure and ‘responsible drinking’, are well-established industry strategies and conceal the contextual influences of youth behaviour. It also serves to reproduce a narrow and industry-friendly conceptualisation of harm reduction limited to reducing harms absent of consumption reduction [93]. Szmigin et al discuss the contradiction inherent to initiatives that seek to change behaviour through familiarisation of units, promotion of ‘sensible drinking’ and warnings against ‘binge drinking’ without understanding or considering the commercial and social contexts [94]. Youth are surrounded by a “culture of intoxication” [95], and calls to consume sensibly are at odds with social norms and behaviours of excessive drinking observed by youth [94]. Such initiatives are discredited by youth as being unrealistic and fail to engage the social nature of youth drinking [94]. As Measham notes “[i]f alcohol education and demand reduction initiatives are not grounded within their appropriate cultural context, a serious credibility gap develops between actual and desirable behaviour”(p262) for consumers, manufacturers, and retailers [96]. Furthermore, as noted by Sussman in relation to youth tobacco programmes [29], there is a contradiction between teaching about the harms of alcohol while portraying it as an adult consumer product: if it is harmful to health why would adults consume it? The contradictory nature of the discourses employed is also evident in the focus on ‘choice’ and decision-making. The tobacco industry established a discourse in which youth ‘choices’ were conceptualised as the focus of intervention. As DiFranza and McAfee [97] have noted, concerning youth smoking there is no ‘choice’, youth should not smoke. The same argument applies to alcohol, yet like tobacco industry-funded materials, those analysed for the purpose of this study focus on choices, and children and youth are not asked to pledge their commitment to not drink.

Our findings echo prior analyses of corporate sponsored youth education programmes which tend to distort the evidence base of harms, problematise peoples’ ‘choices’, focus on peer pressure, and foreclose debate and critical thinking about the role of corporations and wider social and political determinants [5, 6, 11, 18]. For example, there are striking similarities with previous analyses of tobacco industry youth prevention programmes [5, 25, 26, 28, 29, 97, 98]. Main themes can be observed across different types of tobacco industry-generated and funded youth smoking prevention programmes; (a) it is peer pressure and lack of parental role modelling that influence smoking initiation in children, (b) smoking is an adult pursuit, a “forbidden fruit”, and (c) emphasising “the law” as the reason to abstain from smoking [5]. Of note, the “forbidden fruit” appeal was identified by tobacco industry research (“Project 16”) as being an important factor in youth tobacco experimentation [97]. It has been suggested that the alcohol industry pursues this same strategy [99], and was observed in the programmes analysed in this study through articulating alcohol as an enjoyable adult pursuit. Internal industry documents also reveal that the tobacco industry recognised the need to work through reputable third parties to facilitate the delivery of their programmes, given the industry’s declining credibility with the public and policy-makers [5]. Analysis of the alcohol industry-supported resources also reveals divergences from tobacco industry-funded programmes. This is evident in the way the alcohol education recourses focus on teaching ‘responsible’ use of alcohol when of legal age, and acknowledgment of harms (albeit when misused or abused). This is likely in part to reflect the difference in the products and in the social and political contexts in which the programmes are being implemented. The analysis also demonstrates the alignment with education initiatives promoted by major food, beverage, and fossil fuel industries, which similarly fund and promote various education recourses globally, a strategy spanning several decades. These activities serve to support the dissemination of educational recourses that promote personal responsibility, individual-level consumer-based action, shift blame onto children for their ‘poor choices’, and obscure the role of the corporate sponsors in producing and perpetuating the harms and impacts they purport to be teaching children and young people to confront [6, 12].

On face value, these materials may appear to some to be unproblematic as they reflect taken-for-granted notions of individual choices and responsibility, and the benefits of alcohol in moderation [92]. We argue that providers, and those who use them, should scrutinise why these materials attract alcohol industry funding and take seriously the ways in which accepting industry funding jeopardises public health more broadly [100], including how it may serve to forward industry interests by supporting their CSR and political strategies, which in turn can serve to delay evidence-based policies that protect youth. Independent programmes not in receipt of alcohol industry funding can use our analysis to critique their own materials, identifying the ways in which their materials may serve industry interests by maintaining individual-level choice-focused approaches that have become entrenched within the neoliberal health promotion paradigm [101, 102]. Greater understanding is needed of how youth programmes and health education in general serve corporate as opposed to youth interests and the spread of ideas and discourses enabling of corporate interests and power.

The materials analysed failed to acknowledge the lack of evidence regarding the effectiveness of these programmes. As noted for tobacco industry youth programmes [5], when evaluations have been conducted they tend to adopt market research methods with a focus on surrogate outcomes instead of measuring public health outcomes. These evaluations tend to prioritise and promote measures of exposure or levels of awareness, as opposed to actual knowledge and beliefs. As documented by Proctor, the tobacco industry went to great lengths to conflate these concepts [85]. Furthermore, there is no robust, independent evidence that these programmes are effective in preventing or reducing youth drinking and alcohol-related harms.

Interventions identified by systematic review as potentially effective are generic psychosocial and developmental prevention programmes, such as the Unplugged program, Life Skills Training Program, and the Good Behaviour Game [103]. Based on the materials and summaries that are accessible, these programmes do not appear to rely on teaching about units, alcohol production, drinking in moderation, or adopt ‘responsible drinking’ messaging. The Unplugged programme, for example, emphasises the need to stress that alcohol, like tobacco, is a drug, introduces the concept of availability (e.g. in supermarkets) and explains that peer pressure can also serve as a positive influence [104, 105]. The European Drug Addiction Prevention Trial provided recommendations on how to identify an appropriate comprehensive social influences programme for drug prevention, including that the “programme is created or recommended by a not-for profit organization or an authority that has no links with a commercial industry (ie: a tobacco company)”, and that the programme has been robustly evaluated and demonstrated relevant evidence of effectiveness [106]. Similarly, UK Department for Education guidelines stipulate that schools should consider whether a resource “is evidence-based and contains robust facts and statistics” when assessing its acceptability [107]. However, it is important to recognise that a programme may have some limited educational value while simultaneously serving corporate interests in ways that are detrimental to youth and public health more broadly [100, 108]. There is also a lack of evidence on the longer-term impacts of these types of programmes, including future cancer risk behaviours and outcomes, and inequities in these measures, as noted for young adults and their exposure to cancer-risk promoting information and media content [109].

## Wider social context: The commercialisation of education and the ‘neoliberal turn’

The harms of partnering with corporations in the provision of educational programmes are well documented. They are unlikely to be effective (as shown in the case of tobacco [5, 28]), can serve as covert marketing, and are unlikely to be critical of their funders. The materials produced and funded tend to propose solutions that focus on individual ‘choices’ and decision-making, and peer pressure. They deflect away from harmful industry practices, instead conferring upon them a ‘health’ halo [14, 16], with a dominant aim of avoiding tighter restrictions. They distil complex social issues down to often over-simplified dichotomies of ‘good choices’ and ‘bad choices’, and place the burden on children and youth to take responsibility for a major public health issue and their ‘lifestyle choices’, thereby closing down opportunities for critical thinking and debate [6, 11].

These industry practices constitute part of broader developments that have been unfolding in the context of global neoliberal reforms [20]. Schools are subjected to the neoliberal logics of ‘the market’ and privatisation, as well as the growing trend of ‘partnering’ with corporate entities [10, 17, 20]. These phenomena reflect changes in other sectors of society, and reflect the complex nexus of government and corporate interests [20]. Partnered with the widespread delivery of corporatized health education programmes, such shifts restrict teachers and students in their opportunities to question and challenge the status quo and the role of corporations and neoliberal logics in shaping their health [6, 11, 20]. The public health implications of such major structural changes have received far too little attention. Considering the extent of policy and social change that is needed to address major public health issues such as those posed by alcohol harms and the power of the alcohol industry and its supporters, it is concerning that such materials serve to maintain the current order while concealing the potential for other possibilities and ideas.

## Study strengths and limitations

Our study provides an in-depth, critical analysis of three alcohol industry-funded youth education programmes, providing important insights into how these materials serve to maintain and reproduce discourses favourable to corporate interests and shape youth’s knowledge, values and choices in relation to alcohol use and regulation. By adopting a discourse theoretical approach we are able to explore the more nuanced aspects of the texts, placing the findings within the wider social context, thereby extending what would be achieved by undertaking a basic content analysis. The analysis presented here contributes to the emerging literature on the commercial determinants of health, particularly the role of corporations in schools and education, an area in which the activities of the alcohol industry have received limited academic attention. However, the research focused exclusively on the education materials we were able to access at the time of our analysis, and future work is needed to understand the impacts of these programmes on youth, teaching practices, wider societal norms, and the distribution of such impacts. Additionally, content of resources and websites may change with time. We note, for example, that the Smashed Project has, since the period of data collection, redesigned its website and issued an online version of the programme including a teachers resource pack which contains some global statistics and mentions specific cancers, but suggests that the risk is associated with “excessive” alcohol consumption and an information box on the potential damage caused by alcohol lists only mouth and throat cancer [110]. This further supports our calls for greater scrutiny as it raises questions about the governance of the content and review of these resources: why is information added, removed, or modified, and who decides? Similarly, an earlier version of the Talk About Alcohol Teacher Manual and Guidance to the one used in this study briefly mentioned strategic advertising such as the use of ‘alcopops’ and exploitation of Christmas. These points were absent in the more recent 6^th^ edition analysed for this study. More research is needed to monitor and analyse the ways in which these programmes and resources have changed or continue to change over time, and decisions about content changes should be made transparent to the public. Similarly, the funding sources used by, and the associated declarations of funding provided by, the organisations included in this study may change with time.

In the interests of space, we were also unable to explore the use of ‘culture’ blaming or the gendered nature of the presentation of harms within the materials. Further analyses of the parent-facing materials produced by these organisations are also warranted given what has been previously documented about the nature of similar materials produced by the tobacco industry [5, 29, 97]. The aim of our analysis was to analyse resources disseminated with the support of alcohol industry funding, their alignment with previous literature on industry strategies and their role in reproducing the corporate-friendly status quo. In this way, the aim was not to measure the degree to which they differ from those not in receipt of industry funding. Additional research is needed to investigate the extent to which independent programmes align with or diverge from industry generated and funded materials, and the governance of material selection by schools. How the alcohol industry uses its funding of youth programmes for political purposes and with what impact also remains under-researched.

## Conclusions and policy implications

Children and young people have a right to health, and to quality education [111–113]. Industry influence on health education could therefore be approached from a rights perspective. Greater scrutiny of the role of corporations in schools, including health education in receipt of industry funding such as those programmes analysed in our study, is warranted. Such an agenda could afford greater consideration to previous calls for the adoption of a critical pedagogy that teaches about the impacts of corporations on youth and their health, and one that recognises the rights of students and teachers to be aware of, and critique, such practices and the role of corporations in their lives [6, 11, 15, 18]. Saltman and Goodman suggest that one solution is to:

…provide teachers with resources for researching the agendas of the corporations that finance and distribute such products in public schools and museums so that the ideological functions of the curricula can be turned against themselves, and the corporation’s global agendas will be shown as contextualised and centred within the curricula. In this way, students can be shown how their interests and worldviews actually differ from the way their interests and worldviews are constructed in the curricula.(p53) [18]

Powell proposes drawing from diverse forms of culture jamming to support the development of critical pedagogies that could enable teaching about corporate influences [15]. More should be done to draw from effective countering-marketing initiatives, which expose product harms *and* deceptive industry practices, and media literacy programmes, particularly given the impact of alcohol marketing on youth drinking [114–117]. Such practices are yet to be widely adopted and more research is needed to understand the barriers and enablers of uptake and effective implementation.

Most urgently however, our findings call into question the ongoing delivery of such programmes. There are considerable conflicts of interest in the design and delivery of materials provided by industries and the organisations they fund, who have financial interests in the consumption and regulation of the product they are purporting to warn children and youth about. The ongoing exposure of children and young people to such conflicted material needs attention from policymakers, practitioners, teachers, and parents. Independent sources should be reviewed in light of our findings, and materials from sources dependent on industry support should cease being used in schools. Parents and educators should be empowered to identify, critique, and challenge their ongoing use. Policies curtailing the use of such materials in schools should be developed and stated explicitly within government-issued education policy and re-enforced by school boards and education oversight bodies. Finally, alcohol industry-funding and promotion of education-based interventions that shift responsibility for alcohol-related harm to children and young people should not undermine attempts to introduce evidence-based, population-level alcohol control policies that are needed to safeguard children and young people.
